# Aortic root full detachment from the aortic annulus. aortitis role in the formation of a pseudoaneurysm to 3 years of an aortic valve replacement.

**DOI:** 10.1186/1749-8090-10-S1-A312

**Published:** 2015-12-16

**Authors:** R MartinezSanz, R Ávalos, R de la Llana, P Garrido, J Montoto, PC Prada, M Brouard, JL Iribarren, JJ Jiménez, C VaqueroPuerta

**Affiliations:** 1 Complejo Hospitalario Universitario de Canarias, Tenerife, Spain; 2 Universidad de La Laguna, Tenerife, Spain; 3 Universidad de Valladolid, Valladolid, Spain

## Background

Ruptures of the free aortic wall or fistulas to other cardiac structures after aortic valve surgery have been described. They can be due to poor quality of the aortic wall, suture breakage, poor technique or, rarely, aortitis.

## Objective

We present a rare case of complete rupture of the aortic root three years after aortic valve replacement (AVR), with formation of a large pseudoaneurysm, successfully resolved. Enterococcus faecalis RNA was found in aortic wall.

## Patient and Method

A 67 year-old male is brought to the emergency room after suffering syncope. He underwent surgery for AVR 3 years earlier. The computed Tomography (CT) showed a hardly identifiable image at the site of the aortic root. There was a large shift in the origin of the coronary arteries. He underwent emergency surgery.

## Results

Redo open-heart surgery was performed, using femoral cannulation, mild hypothermia at 28°C, and circulatory arrest during 4 minutes, just to open and inspect the aorta. A big cavity acting as the aortic root, with irregular contour was observed. The floor of the cavity was the aortic prosthesis, the roof the beginning of true aortic root including both *coronary ostia *and the walls were formed by the roof of the left atrium, the main pulmonary artery, superior vena cava, right pulmonary artery and the rests of fibrotic and adhered pericardium. Aortic prosthesis seemed normofunctional, but the walls of the ascending aorta and aortic root were inflamed. A Bentall-De bono technique was performed. An Enterococcus faecalis was identified in the aortic wall by polymerase chain reaction. He was given six weeks of antibiotic therapy.

## Conclusions

Aortitis after AVR may cause complete rupture of the aortic root by its own detachment, creating a pseudoaneurysm, which might rupture. The preoperative diagnosis is not easy as well the surgical treatment.

## Consent

Written informed consent was obtained from the patient for publication of this abstract and any accompanying images. A copy of the written consent is available for review by the Editor of this journal.

